# Comparing walking with knee-ankle-foot orthoses and a knee-powered exoskeleton after spinal cord injury: a randomized, crossover clinical trial

**DOI:** 10.1038/s41598-022-23556-4

**Published:** 2022-11-09

**Authors:** Antonio Rodríguez-Fernández, Joan Lobo-Prat, Rafael Tarragó, Diego Chaverri, Xavier Iglesias, Lluis Guirao-Cano, Josep M. Font-Llagunes

**Affiliations:** 1 Biomechanical Engineering Lab, Deparment of Mechanical Engineering and Research Center for Biomedical Engineering, Universitat Politècnica de Catalunya, 08028 Barcelona, Spain; 2 Institut de Recerca Sant Joan de Déu, 08950 Esplugues de Llobregat, Spain; 3 ABLE Human Motion, 08028 Barcelona, Spain; 4 Grup de Recerca en Ciències de l’Esport INEFC Barcelona, Institut Nacional d’Educació Física de Catalunya, Universitat de Barcelona, Barcelona, 08038 Spain; 5 Rehabilitation Service, Asepeyo Hospital Barcelona, 08174 Barcelona, Spain

**Keywords:** Rehabilitation, Spinal cord diseases, Biomedical engineering

## Abstract

Recovering the ability to stand and walk independently can have numerous health benefits for people with spinal cord injury (SCI). Wearable exoskeletons are being considered as a promising alternative to conventional knee-ankle-foot orthoses (KAFOs) for gait training and assisting functional mobility. However, comparisons between these two types of devices in terms of gait biomechanics and energetics have been limited. Through a randomized, crossover clinical trial, this study compared the use of a knee-powered lower limb exoskeleton (the ABLE Exoskeleton) against passive orthoses, which are the current standard of care for verticalization and gait ambulation outside the clinical setting in people with SCI. Ten patients with SCI completed a 10-session gait training program with each device followed by user satisfaction questionnaires. Walking with the ABLE Exoskeleton improved gait kinematics compared to the KAFOs, providing a more physiological gait pattern with less compensatory movements (38% reduction of circumduction, 25% increase of step length, 29% improvement in weight shifting). However, participants did not exhibit significantly better results in walking performance for the standard clinical tests (Timed Up and Go, 10-m Walk Test, and 6-min Walk Test), nor significant reductions in energy consumption. These results suggest that providing powered assistance only on the knee joints is not enough to significantly reduce the energy consumption required by people with SCI to walk compared to passive orthoses. Active assistance on the hip or ankle joints seems necessary to achieve this outcome.

## Introduction

Most people with a spinal cord injury (SCI) have permanent paralysis of the lower limbs and lose their ability to stand and walk. This functional limitation often leads to wheelchair dependency and sedentary behaviour that increases the risk of developing secondary health conditions^1–3^. Therefore, recovering the ability to stand and walk independently can have numerous health benefits for people with SCI^4–6^, and has been a priority in rehabilitation medicine^7^.

Passive gait orthoses, such as knee-ankle-foot orthoses (KAFOs), are often used in clinical practice and also prescribed for home use after discharge. Orthotic long-term use after rehabilitation in people with SCI is varied (rejection rate ranges from 15 to 85%)^8–15^, and inversely related to the level of injury (LOI) and age^16^. The use of passive gait orthoses for assisting functional mobility is controversial as their use requires a very high energy expenditure, which often leads to exhaustion after a few minutes of walking. In fact, Merkel et al.^17^ found that energy cost of walking with KAFOs in SCI patients with an injury at low or mid thoracic level (from T4 down) was about eight times higher than for normal walking. Excessive energy consumption has been reported as one of the main problems leading to orthoses’ rejection^8–15^. In addition, when using passive gait orthoses, people with SCI adopt unusual strategies to throw the leg forward, such as the swing-through gait or hip compensatory movements (i.e., hip hiking or circumduction)^18–22^; and use their upper extremities to generate forward propulsion^23^, which can lead to musculoskeletal injuries or shoulder pain^24,25^.

In response to the aforementioned limitations of passive gait orthoses, wearable, robotic exoskeletons are emerging as an alternative technology for assisting gait in people with SCI as they may augment gait efficiency, allow for a more physiological gait, and provide safer walking compared to KAFOs^26,27^. These improvements could give people with SCI the possibility of carrying out activities of daily living, walking outdoors, and socializing easier within varied environment^5,28,29^. However, there is a lack of evidence that supports the superiority of wearable exoskeletons over KAFOs as a gait assistive device. While some studies have focused on evaluating the effort needed to use powered exoskeletons^30–35^ or passive orthoses^13,17,36^, just a few aimed to compare between orthotic and exoskeleton assisted gait^26,37–39^. Note that in this last group of studies the energy efficiency was indirectly estimated by the physiological cost index (PCI)^26,37,38^, i.e., dividing the difference between walking and resting heart rate by velocity, and only the study by Kwon et al.^39^ used oxygen consumption ($VO_{2}$) to measure energy efficiency. Moreover, none of them compared gait kinematics. All the aforementioned studies found that energy efficiency was higher when walking with the exoskeleton than with the passive orthotic device. Nevertheless, all these studies used exoskeletons that provided (at least) powered hip and knee assistance, and it remains unknown how gait performance and energy efficiency of walking with KAFOs would compare to walking with a wearable exoskeleton that only provides knee assistance.

Knee-powered lower limb exoskeletons can be seen as an attractive solution for assisting functional mobility in people with SCI, considering that they provide a more physiological knee motion pattern compared to KAFOs, which may eliminate the side effects caused by the unnatural gait pattern when using KAFOs (e.g., pain, injuries, excessive physical exertion, and activity avoidance^40^). Likewise, knee exoskeletons can be noticeably lighter^41^ and easier to operate than hip-knee-powered exoskeletons, mainly due to their reduced size and simplicity. Nonetheless, the high positive mechanical energy provided by the hip during gait and gait initiation^42,43^ raises uncertainty as to whether knee-only assistance would be sufficient to decrease the physical demand involved in walking with KAFOs.

The purpose of this study was to compare the use of a knee-powered lower limb exoskeleton (i.e., the ABLE Exoskeleton) against conventional KAFOs in people with SCI, specifically aiming to answer the following research questions: (1) Is gait performance, in terms of kinematics and spatiotemporal parameters, better with the ABLE Exoskeleton than with KAFOs? (2) Does the ABLE Exoskeleton reduce the metabolic cost of walking compared to KAFOs? and (3) What is the participants’ level of satisfaction with both devices? To answer these questions, we conducted a randomized, crossover clinical trial where we assessed gait kinematics, spatiotemporal parameters and energy expenditure during standardized clinical tests. We hypothesized that walking with the ABLE Exoskeleton would result in a more physiological gait pattern (i.e., reduction of compensatory gait strategies), which would lead to an improvement of the spatiotemporal parameters, a reduction in the metabolic cost, and a higher user satisfaction compared to walking with the KAFOs.

## Methods

### Study design

This study was a randomized, single-center, crossover clinical trial (see Supplementary Fig. 1) that compared walking with KAFOs (i.e., the standard of care for verticalization and ambulation outside the clinical setting in people with SCI) against walking with a knee-powered bilateral lower limb exoskeleton (i.e., the ABLE Exoskeleton). The clinical trial was performed at Asepeyo Sant Cugat Hospital (Barcelona, Spain), a center specialized in SCI, from February to August 2021. The total duration of each patient’s training was approximately 12 weeks. The study conformed to the principles of the Declaration of Helsinki (revised version 2013), the ISO 14155:2011, and the European Regulation MDR 2017/745 on medical devices. The clinical trial was approved by the responsible ethics committee (CEIm Grupo Hospitalario Quirónsalud-Catalunya; study code: 2020/157-REH-ASEPEYO) and the national competent authority (Spanish Agency of Medicines and Medical Devices (AEMPS), EUDAMED: CIV-ES-21-01-035724). The study protocol was first registered at ClinicalTrials.gov on 22/04/2021 (NCT04855916).

### Participants

The inclusion criteria for this study (see Supplementary Table 1) were designed to recruit patients with SCI at the investigational site. Eleven outpatients with chronic (i.e., time since injury more than one year ago) motor-complete SCI (AIS grade A/B) were assessed for eligibility in this study. One patient was excluded for not meeting the inclusion/exclusion criteria and ten were enrolled (see Supplementary Fig. 1) and completed the full protocol (Table 1). The neurological level of injury of the participants ranged from T4 to T12. On average, participants were $44.10 \pm 5.93$ years old and mostly male (90%). Seven participants had a traumatic SCI while three had a non-traumatic SCI. All participants had their own KAFOs at home and had previous experience using them, which was corroborated through a test where participants had to walk 5 m with KAFOs, little therapist assistance, and the aid of a walker. Moreover, three of them had previous experience using other wearable lower limb exoskeletons. Participants were assigned through simple randomization to one of two groups: KAFO or ABLE. The principal investigator blindly chose one of the 20,000 lines of a book of random numbers^44^. The ten first numbers of the chosen line (from left to right) were used to allocate each of the participants to one of the two groups following the order of enrollment: even numbers assigned participants to the ABLE group and odd numbers assigned participants to the KAFO group. Prior to data collection, each patient provided written informed consent to take part in the study.

**Table 1:** Patient demographics.

Patient	Age (years)	Gender	Time since injury (years)	Level of injury	ASIA scale	Height (cm)	Weight (kg)	Starting device
P1	39	M	11	T4	A	176	80	K
P2	46	F	7	T4	A	168	70	K
P3	44	M	12	T4	A	170	74	A
P4	40	M	21	T6	A	174	72	A
P5	55	M	5	T8	A	170	80	A
P6	47	M	2	T11	A	183	77	K
P7	33	M	8	T12	A	169	84	K
P8	48	M	10	T8	B	173	75	A
P9	46	M	23	T11	A	185	98	A
P10	43	M	6	T10	B	173	71	K

M: Male, F: Female, K: KAFOs, A: ABLE Exoskeleton.

### Study protocol

**Figure 1:**
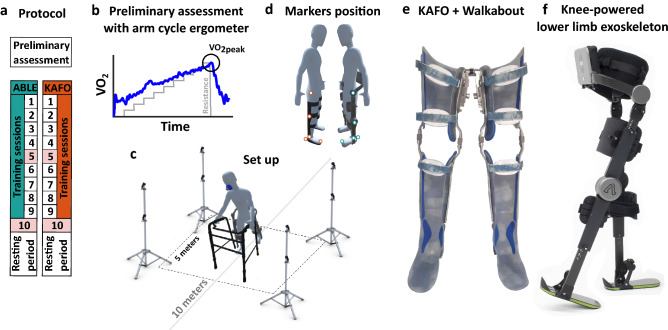
(**a**) Study protocol. (**b**) Representation of the data obtained from the GXT performed with an arm cycle ergometer. (**c**) Set up representation for the 6 MWT. (**d**) Marker protocol: Markers were placed at the trochanter, lateral side of the knee, lateral side of the ankle, hallux, and heel (achilles attachment). (**e**) Knee-ankle-foot orthoses with Walkabout. (**f**) Knee-powered lower limb exoskeleton; i.e., the ABLE Exoskeleton (ABLE Human Motion S.L., Barcelona, Spain).

The study consisted of 10 sessions (2 sessions per week, during 5 weeks) of 90-min duration (Fig. 1a): 8 overground gait training sessions (sessions 1 to 4 and 6 to 9) plus 2 evaluation sessions (sessions 5 and 10). Note that the present publication focused on analyzing the results from the final evaluation session (session 10). The primary clinical outcome measure was the metabolic cost of walking with both devices that was measured through gases exchange. Secondary outcome measures included gait kinematics, spatiotemporal parameters, and psychoshocial impact and user satisfaction questionnaires.

Participants spent a minimum of 30 min per training session doing sit-to-stand and stand-to-sit transitions, and standing and walking exercises using one of the two devices and the aid of a walker. There was a 2-week resting period between the final evaluation session and the first training session with the crossed-over device. At the beginning of the study, before using any device, there was a first visit that included a preliminary assessment consisting of a graded exercise test (GXT) with an arm cycle ergometer (Fluid E920 Medical UBE, First Degree Fitness, The Netherlands) (Fig. 1b). After a resting minute, participants started to cycle with a resistance of 15 W at 60–70 rpm, and every minute a 5 W resistance was added until the participants were no longer able to continue cycling. All participants cycled for at least 9 min and we recorded the peak oxygen uptake ($VO_{2peak}$).

To evaluate our hypotheses, participants performed three standardized clinical tests during session 10 using one of the two devices (Fig. 1a) with the help of a walker: the timed up and go (TUG), the 6-min walking test (6 MWT), and the 10-min walking test (10 MWT). For the TUG, participants remained in a sitting position for 3 min after which the test started. The test consisted of walking back and forth in a 3-m pathway, starting and finishing seated on a chair. After the test, there was a 3-min cool down period. This process was repeated twice with a 5-min break between trials to recover from fatigue. The time needed to complete each TUG was recorded. The 6 MWT was conducted after a resting period of 15–20 mins after the cool down period of the second TUG. During this time, reflective markers were placed on the participants to capture gait kinematics using an optical system (see section Data Collection). Before the 6 MWT, participants were asked to rest in a sitting position for 3 min, and 30 s before finishing the resting period, they were asked to stand up and be ready to start. During the test, participants walked in a 10-m pathway at a self-selected speed. Participants were allowed to rest and stop the test as necessary. In that last case, the distance covered during that time was recorded. Resting and turning times were included in the 6 MWT. Walking speed was determined by dividing the total walking distance by the total time walking (m/min). During the 6 MWT, spatiotemporal parameters, gait kinematics, and energy consumption were measured. The 10 MWT was measured during the first 10 m of the 6 MWT. Time, gait speed, and cadence were calculated from the middle-placed 6 m (the first and last 2 m were taken for acceleration and deceleration, respectively). After the final evaluation session, participants filled out the Quebec User Evaluation of Satisfaction with Assistive Technology (QUEST 2.0)^45^ and the Psychosocial Impact of Assistive Devices Scale (PIADS)^46^ questionnaires to evaluate users’ satisfaction and the psychosocial impact that the training with the corresponding device may have had.

### Gait assistive devices

#### Knee-ankle-foot orthosis

Knee-ankle-foot orthoses are passive leg braces customized to each individual that provide stability by locking the knee joint at full extension and the ankle at the neutral position^16^. There are several common indications for using KAFOs, but muscle weakness and paralysis are the most frequent. In general, it consists of an orthopedic thermoplastic cast of the thigh and shank segments attached to metal bars that are connected to a foot insole.

In this study, all the participants used their own KAFOs made of thermoplastic that had been prescribed from the investigational site (Fig. 1e), and intended for upright ambulation at home. Note that 8 out of the 10 participants used the KAFOs in combination with a multiaxial subperineal hip joint (also known as Walkabout joint) that stabilizes stance and provides a reciprocating gait. The average mass of the KAFOs including the Walkabout unit was 3.16 kg (ranging from 2.5 to 3.7 kg).

#### ABLE Exoskeleton

The ABLE Exoskeleton (ABLE Human Motion S.L., Barcelona, Spain) is a knee-powered lower limb exoskeleton (Fig. 1f) that actively assists with sit-to-stand, standing upright, walking, and stand-to-sit, and is composed of 5 modules: shank (left and right), thigh (left and right), and lumbar. Regarding walking assistance, the ABLE Exoskeleton provides knee flexion-extension assistance during the swing phase following a predefined knee joint angle trajectory (Fig. 3b). During the stance phase, the knee remains fully extended. A passive hip joint allows hip flexion-extension and constrains hip abduction-adduction and internal-external rotation. The ankle joint is fixed keeping the foot perpendicular to the shank. The exoskeleton can be adjusted to the length of the shank and thigh segments and the hip-width of each participant. The battery is placed on the lumbar module and the total mass of the ABLE Exoskeleton is 9.80 kg.

The initiation of each step can be triggered either (1) manually by the therapist using the buttons in the lumbar module or (2) automatically by the step intention of the user, detected through the thigh angular velocity and measured with an inertial measurement unit (IMU). Additionally, the ABLE Exoskeleton presents two operation modes to transition between states (i.e., sitting-to-standing, standing-to-walking, walking-to-standing, and standing-to-sitting) based on the user’s expertise. For beginners, the exoskeleton can be controlled by the therapist using the lumbar buttons. For more advanced users, state transitions can be controlled by the users themselves through a remote controller attached to the walker.

Step parameters are configured using the mobile app ABLE Care (ABLE Human Motion S.L., Barcelona, Spain) and define the shape of the knee angular trajectory during the swing phase. Parameters for peak knee flexion, swing time, and flexion-extension time ratio (i.e., time displacement of the peak knee flexion) can be tuned by the therapists in order to achieve sufficient foot clearance and maximize the step length of the users. The ABLE Care is also used to monitor user performance (i.e., gait speed, distance, number of steps, step length, and stance time ratio between left and right leg).

Finally, note that the ABLE Exoskeleton should not be mistaken for stance-control KAFOs (SCKAFOs). Despite that both devices lock the knee joint in a fully extended position during the stance phase, the SCKAFOs are a particular case of KAFOs that allow for free knee motion during the swing phase; while the ABLE Exoskeleton actively assists the knee flexion-extension motion during the swing phase.

### Data collection

Kinematic data were recorded using reflective markers placed at the trochanter, lateral side of the knee, lateral side of the ankle, hallux, and heel (achilles attachment) of each leg (Fig. 1d). Markers were attached to the structure of the gait assistive device when anatomical landmarks were blocked by the device (KAFO: knee and ankle; ABLE: trochanter, knee, and ankle). The three-dimensional marker positions were recorded at a sampling rate of 100 Hz with 10 motion capture cameras (OptiTrack Flex V100:R2, NaturalPoint Inc., Corvallis, OR, USA), which covered 5 m of the middle part of the 10-m pathway (Fig. 1c). Metabolic cost was assessed with indirect calorimetry using a portable gas analysis system (K4b$^{2}$, COSMED Wearable Metabolic Systems, Italy) during both the 6 MWT and the 3 min before the test.

The level of assistance (LoA) needed for walking with the devices was recorded in each training session. The LoA was reported by the clinical staff using a rating scale adapted from the Functional Independence Measure (FIM)^47^. The only difference with the FIM was that the *modified independence* score (i.e., patient requires use of a device, but no physical assistance) was removed, since all the participants in this study used an assistive device for walking. The modified FIM used in this study and the LoA needed for each participant to complete the TUG, the 6 MWT, and the 10 MWT in session 10 can be found in the Supplementary Fig. 2.

### Data analysis

Marker data were low-pass filtered at 6 Hz and gait events (heel strike and toe-off) were detected manually (since gait pattern was unpredictable in most cases) using toe and heel marker trajectories in the sagittal plane.

Circumduction was defined as the maximum lateral difference of the ankle marker between stance and swing phase, similar to the study by Awad et al.^48^. Weight shifting (i.e., actively shifting weight towards the leading leg before swing phase of the trailing leg) was indirectly measured by the relative position of the projected center of mass (PCOM) with respect to the middle point of the leading foot at toe-off.

The circuity index was used to measure the path efficiency of the PCOM during the gait cycle in the transverse plane. The circuity index, which is defined as the ratio between the distance covered by the PCOM trajectory and the euclidean distance between start and end points, has been used in a variety of contexts^49^. Note that the center of mass (COM) was estimated as the middle point between the markers placed at the trochanters, and the middle point of the foot was estimated as the middle point between the heel and toe markers.

For the analysis of gases, the data collected during the 6 MWT were low-pass filtered at 0.04 Hz (following the recommendations from Robergs et al.^50^) and interpolated from breaths to seconds. Filtered signals were averaged ($VO_{2avg}$) over the last 2 min to estimate the steady-state energetic expenditure, when a plateau had been reached. The levels for physical intensity were defined as light (37–45 $\%VO_{2peak}$), moderate (46–63 $\%VO_{2peak}$), vigorous (64–91 $\%VO_{2peak}$), and maximal (> 91 $\%VO_{2peak}$)^51^. Walking efficiency was estimated by the Metabolic Cost of Transport (*MCoT*, [ml/kg/m]) using Eq. (1). We used the definition proposed by Martini et al.^52^, which was used to determine gait efficiency in elderly people when using a robotic hip exoskeleton. Walking efficiency refers to the amount of oxygen consumed per 1 kg of body weight to walk a 1 m distance^53,54^.

1 $$\begin{aligned} MCoT = \frac{\int _{t_{ini}}^{t_{end}} ({\dot{V}}O_{2} - {\dot{V}}O_{2B})\,dt}{L} \end{aligned}$$

where ${\dot{V}}O_{2}$ [ml/s/kg] is the filtered oxygen uptake rate, ${\dot{V}}O_{2B}$ [ml/s/kg] is the average baseline oxygen uptake rate recorded during the second minute of the resting period, $t_{ini}$ [s] is the walking start time, $t_{end}$ [s] is the walking final time, and *L* [m] is the distance covered during the test. The software used for all data analysis was MATLAB (MATLAB R2021b, The MathWorks Inc., Natick, MA, USA).

Regarding the questionnaires, only the items of the QUEST 2.0 related to user satisfaction with the assistive devices were assessed (i.e., service delivery items were omitted). The analysis of the PIADS was done following the instructions of the manual proposed by Day and Jutai^46^.

### Statistical analysis

The sample size of the present clinical investigation (i.e., 10 patients) was set taking into account previous clinical studies that also had the objective of comparing the performance of a robotic exoskeleton against the standard of care for gait assistance in people with SCI (i.e., KAFOs)^26,37–39^, or the objective of measuring the metabolic cost^31,32,34^ or effort^30^ required to walk with a robotic exoskeleton.

For each metric of interest related to kinematics and spatiotemporal parameters, we divided the data into gait cycles (i.e., from heel strike to heel strike of the same leg), and calculated the average value for each metric, patient, and device. The number of gait cycles used per participant for the kinematics and spatiotemporal analysis depended on the distance covered during the 6 MWT. On average, we used $9.50 \pm 6.68$ gait cycles per participant (range = [2, 24]).

The same approach was conducted for the metabolic cost variables, obtaining an average value for each metric, patient, and device. Statistics were applied to the resulting mean and standard deviation obtained through the average values of the 10 participants. To evaluate differences between devices, paired 2-tailed t-tests or Wilcoxon signed-rank tests were used based on the distribution’s normality quantified by the Kolmogorov-Smirnov statistic. Correlation between LOI and variables of interest was measured with the Spearman correlation coefficient ($\rho$). The level of significance was set to $\alpha$ = 0.05. Statistical analyses were carried out using R version 4.2.0.

## Results

All study participants but P10, who missed one training session with the ABLE Exoskeleton, completed the protocol as scheduled. No serious adverse events were reported during the study.

When using the robotic device, 9 out of the 10 participants did the final test with the automatic step initiation trigger and used the remote controller to control state transitions. The complete set of step parameters of the ABLE Exoskeleton that was used by each of the participants during session 10 can be found in the Supplementary Table 2. The average LoA provided by the therapist to the participants was slightly higher for the ABLE group compared to the KAFO group, yet this difference was not statistically significant (see Supplementary Fig. 2). A table with all the results (mean ± standard deviation, % difference between devices, and p value) from this study can also be found in the Supplementary Table 3.

### Clinical evaluations

**Figure 2:**
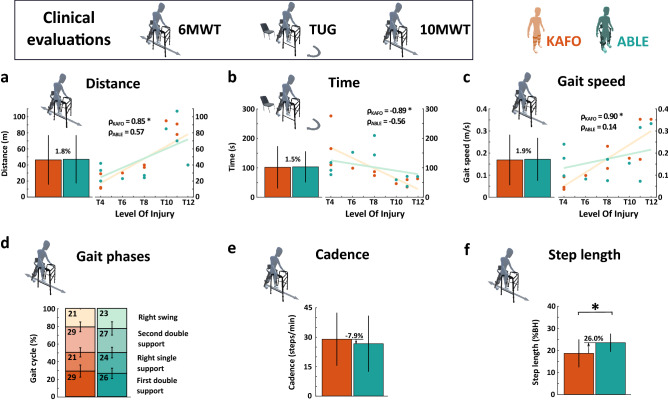
(**a**) Distance covered during the 6 MWT. (**b**) Time needed to complete the TUG. (**c**) Gait speed during the 10 MWT. (**d**) Gait phases during the 6 MWT. (**e**) Cadence during the 10 MWT. (**f**) Step length in % of body height (%BH) during the 6 MWT. Bar plots show the mean and standard deviation from all the participants. The correlation was measured with the Spearman correlation coefficient ($\rho$). Stars (*) indicate statistically significant differences (*p* $< 0.05$).

Figure 2a–c show the distance covered during the 6 MWT, time needed to complete the TUG, and gait speed during the 10 MWT. No significant differences were found between the two groups (i.e., KAFO and ABLE) for the outcome metrics of the standardized clinical tests (6 MWT: *p* = 0.910; TUG: *p* = 0.359; 10 MWT: *p* = 0.492). Linear regression analysis between the outcome metrics of the standardized clinical tests and the level of injury revealed significant, strong correlations for the KAFO group (6 MWT: $\rho _{KAFO}$ = 0.85, *p* = 0.002, 95% CI [0.46, 0.96]; TUG: $\rho _{KAFO}$ = − 0.89, *p* $< 0.001$, 95% CI [− 0.97, − 0.59]; 10 MWT: $\rho _{KAFO}$ = 0.90, *p* $< 0.001$, 95% CI [0.63, 0.98]). In contrast, correlations for the ABLE group were low to mild and not statistically significant (6 MWT: $\rho _{ABLE}$ = 0.57, *p* = 0.087, 95% CI [− 0.10, 0.88]; TUG: $\rho _{ABLE}$ = − 0.56, *p* = 0.095, 95% CI [− 0.87, 0.11]; 10 MWT: $\rho _{ABLE}$ = 0.14, *p* = 0.696, 95% CI [− 0.54, 0.71]).

### Spatiotemporal parameters

Step length (measured during the 6 MWT) revealed a significant average increase of 26.0% when walking with the ABLE Exoskeleton compared with KAFOs (KAFO: $18.60 \pm 6.31$% of body height (%BH); ABLE: $23.43 \pm 4.10$% BH; *p* = 0.037; Figure 2f). Gait phases (measured during the 6 MWT) did not show significant differences between the two devices (first double support: *p* = 0.275; right single support: *p* = 0.232; second double support: *p* = 0.492; right swing: *p* = 0.625; Figure 2d). Finally, albeit not statistically significant, walking cadence (measured during the 10 MWT) showed an average reduction of a 7.9% when walking with ABLE compared with KAFOs (KAFO: $28.91 \pm 13.51$ steps/min; ABLE: $26.62 \pm 14.28$ steps/min; *p* = 0.695; Figure 2e).

### Gait kinematics

#### Gait pattern

**Figure 3:**
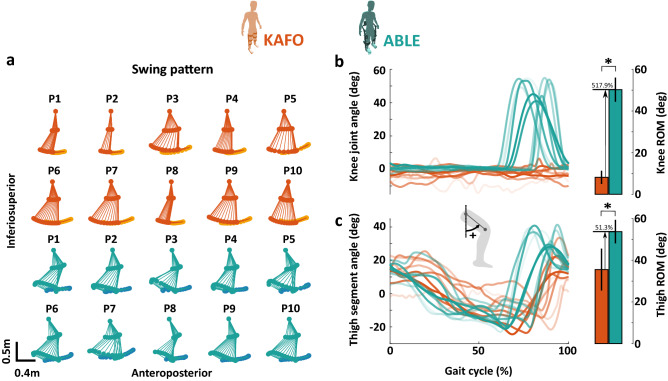
(**a**) Average swing pattern of the leg in the sagittal plane of each participant and device during the 6 MWT. The toe marker is shown with a different color (KAFO: light orange, ABLE: blue). (**b**) Representative knee flexion/extension angle of each participant during the gait cycle, and a bar plot showing the average and standard deviation from all the participants’ ROM. (**c**) Representative thigh segment angle in sagittal plane of each participant during the gait cycle, and a bar plot showing the average and standard deviation from all the participants’ ROM. The knee joint and thigh segment angles were measured during the 6 MWT. Stars (*) indicate statistically significant differences (*p* $< 0.05$).

The swing pattern of the leg in the sagittal plane, the knee joint angle and the thigh segment angle trajectories and ROM of each participant for both devices during the 6 MWT are presented in Fig. 3. Compared to walking with the KAFOs, walking with the ABLE Exoskeleton significantly increased the ROM of both knee joint angle (KAFO: $8.13 \pm 3.16$ degrees; ABLE: 50.25 ± 5.81 degrees; *p* = 0.002) and thigh segment angle (KAFO: 35.54 ± 10.08 degrees; ABLE: $53.77 \pm 5.64$ degrees; *p* = 0.004) by 517.9% and 51.3%, respectively.

#### Compensatory movements

**Figure 4:**
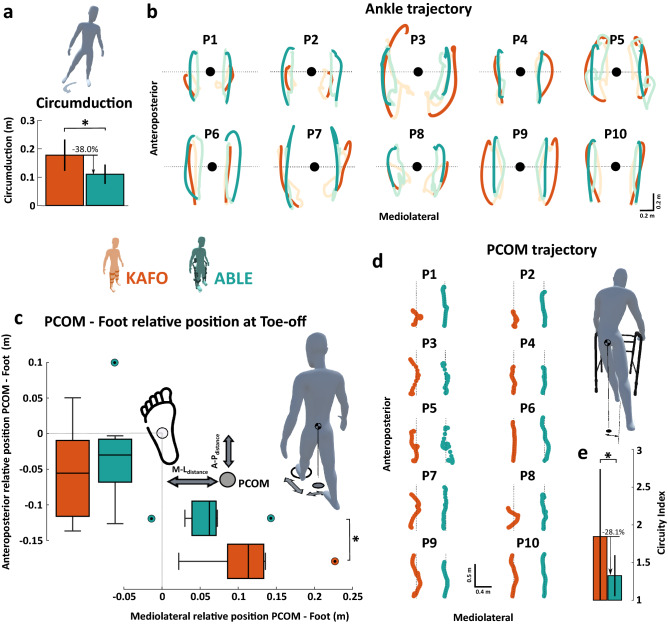
(**a**) Circumduction during the 6 MWT. Circumduction was defined as the maximum lateral difference of the ankle marker between stance and swing phase. Bars show the mean and standard deviation from all the participants. (**b**) The average ankle marker trajectory with respect to the PCOM in the transverse plane during the gait cycle for each participant. Black circles represent the PCOM, measured as the middle point between trochanters. (**c**) Box plot of the relative position of the PCOM with respect to the center of the leading foot at toe-off in both anteroposterior and mediolateral directions. Statistical analysis was done using the mean values using each device. (**d**) Trajectories of the PCOM (big circle) in the transverse plane during an average gait cycle for each participant. Dashed lines show the straight path to follow while walking. (**e**) Circuity index mean and standard deviation using each device. The circuity index is defined as the ratio of the total distance covered by the PCOM trajectory to the euclidean distance between its first and last position in the transverse plane during a gait cycle. Data were recorded during the 6 MWT. Stars (*) indicate statistically significant differences (*p* $< 0.05$).

Figure 4b shows a top view of the average ankle trajectories (i.e., projected in the transverse plane) of each participant when walking with KAFOs and the ABLE Exoskeleton. We found that walking with the ABLE Exoskeleton significantly reduced circumduction by 38.0% on average (Fig. 4a), compared to walking with KAFOs (KAFO: $0.18 \pm 0.06$ m; ABLE: 0.11 ± 0.03 m; *p* = 0.014).

#### Weight shifting

Figure 4c shows the results of the relative position of the PCOM with respect to the center of the leading foot in both anteroposterior and mediolateral directions at toe-off. We found a significant difference in the position of the PCOM relative to the leading foot in the mediolateral direction (KAFO: 0.11 ± 0.05 m; ABLE: $0.06 \pm 0.04$ m; *p* = 0.010) with a reduction of 48.9% on average when walking with the ABLE Exoskeleton, but not in the anteroposterior direction (KAFO: $0.05 \pm 0.07$ m; ABLE: 0.03 ± 0.06 m; *p* = 0.322), where the position of the PCOM relative to the leading foot reduced by 40.2% on average. The distance between PCOM and the leading foot was significantly shorter when walking with the ABLE Exoskeleton with a reduction of 29.3% on average (KAFO: $0.16 \pm 0.04$ m; ABLE: $0.12 \pm 0.03$ m; *p* = 0.006).

Figure 4d shows the trajectories of the PCOM in a top view during an average gait cycle using both devices. The analysis of the circuity index revealed that walking with the ABLE Exoskeleton the PCOM trajectory was significantly closer to the ideal straight path by 28.1% on average, compared to walking with KAFOs (Fig. 4e). Note that a circuity index of 1 indicates that the PCOM trajectory would follow a perfect straight line (KAFO: $1.84 \pm 0.90$; ABLE = $1.33 \pm 0.27$; *p* = 0.049).

### Gait efficiency

**Figure 5:**
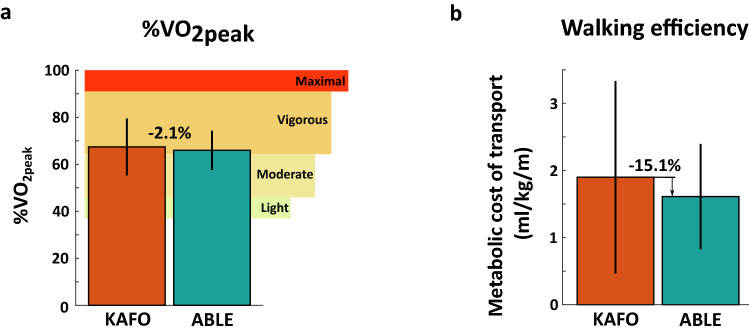
(**a**) Percentage of the peak oxygen uptake ($\%VO_{2peak}$) for each device, calculated by dividing the $VO_{2avg}$ by the $VO_{2peak}$ obtained from the maximal graded exercise during the preliminary assessment. The $VO_{2avg}$ is the average oxygen consumption over the last 2 min of the 6 MWT. The $VO_{2peak}$ is the maximum value recorded during the maximal graded exercise. Levels of physical intensity were defined as light (37–45 $\%VO_{2peak}$), moderate (46–63 $\%VO_{2peak}$), vigorous (64–91 $\%VO_{2peak}$), and maximal (>91 $\%VO_{2peak}$)^51^. (**b**) Walking efficiency during the 6 MWT for each device estimated by the Metabolic Cost of Transport (*MCoT*), according to the equation used by Martini et al.^52^. Walking efficiency refers to the amount of oxygen consumed per 1 kg of body weight to walk a distance of 1 m.

Figure 5 shows the results of the walking efficiency in terms of metabolic cost of transport (*MCoT*) and the intensity of physical activity as a percentage of peak oxygen uptake ($\%VO_{2peak}$) during the 6 MWT. The $\%VO_{2peak}$ indicated that, on average, participants walked at vigorous intensity level (i.e., > 64 $\%VO_{2peak}$) with both devices. No significant differences between the ABLE Exoskeleton and the KAFOs were found in terms of the *MCoT* (KAFO: $1.90 \pm 1.43$ ml/kg/m, ABLE: $1.61 \pm 0.78$ ml/kg/m, *p*= 0.528) nor in terms of the $\%VO_{2peak}$ (KAFO: 67.39 ± 12.12 $\%VO_{2peak}$; ABLE: $65.98 \pm 8.34$ $\%VO_{2peak}$; *p* = 1).

Table 2 shows a comparison of the results of the present study with previous literature on the metabolic cost of walking, measured through oxygen consumption, using exoskeletons and passive orthotic devices in people with SCI. Note that all the exoskeletons used in previous studies and shown in Table 2 had, at least, hip and knee actuation. We found that previous studies reported, on average, that walking with the (hip-knee-powered) exoskeletons was 35% more efficient (i.e., metabolic cost of transport) and 21.95% more effortless (i.e., $\%VO_{2peak}$) compared to our results with the (knee-powered) ABLE Exoskeleton. Furthermore, Kwon et al.^39^, the only study that measured oxygen consumption and compared with KAFOs, showed a reduction of 23.7% in the $VO_{2avg}$ during the 6 MWT when walking with the ReWalk Exoskeleton (ReWalk Robotics, Yokneam Illit, Israel) compared to walking with the KAFOs (ReWalk: $9.0 \pm 2.1$ ml/kg/min, KAFO-Kwon: $11.8 \pm 1.8$ ml/kg/min). Interestingly, the difference between the $VO_{2avg}$ when walking with the ABLE Exoskeleton and walking with passive orthoses from previous studies is higher (6.31%) than the difference found between the ABLE Exoskeleton and the KAFOs used in the present study (1.25%).

**Table 2:** Metabolic cost of walking with wearable exoskeletons and passive orthoses in previous studies and the present study.

Study	Device	Actuation	Number of subjects	Level of injury	${\mathbf {VO_2avg}}$ (ml/kg/min)	${\mathbf {\%VO_2peak}}$	Metabolic cost of transport (ml/kg/m)	${\mathbf {\Delta VO_2}}$ $^1$ (ml/kg/min)	${\mathbf {MET_{sci}}}$ $^2$
This study	ABLE	K	10Co	T4–T12	$13.62 \pm 1.96$	$65.98 \pm 8.34$	$1.61 \pm 0.78$	$10.22 \pm 1.75$	$5.04 \pm 0.73$
Kwon et al.^39^	ReWalk	HK	10Co	T4–T11	$9.00 \pm 2.10$ (− 33.92%)	–	–	–	3.33 * (− 33.93%)
Evans et al.^32^	Indego	HK	5Co	T6–T12	$9.50 \pm 0.80$ (− 30.25%)	$51.5 \pm 9.90$ (− 21.95%)	–	–	$3.50 \pm 0.30$ (− 30.56%)
Park et al.^53^	H-MEX	HK	8Co + 2In	C6–L1	$9.40 \pm 1.90$ (− 30.98%)	–	$1.2 \pm 0.4$ (− 25.47%)	$6.2 \pm 2.10$ * (− 39.33%)	$3.50 \pm 0.70$ (− 30.56%)
Asselin et al.^31^	ReWalk	HK	7Co + 1In	T1–T11	$11.20 \pm 1.70$ (− 17.77%)	–	–	$7.70 \pm 1.75$ * (− 24.66)	–
Knezevic et al.^55^	ReWalk	HK	5Co	T1–T11	$12.73 \pm 2.30$ (− 6.53%)	–	$0.87 \pm 0.85$ (− 45.96%)	$8.88 \pm 2.38$ * (− 13.11%)	–
Average of studies using exoskeletons	–	–	–	–	10.37 (− 23.86%)	$51.5 \pm 9.90$ (− 21.95%)	1.04 (− 35.40%)	7.59 (− 25.73%)	3.44 (− 31.75%)
This study	KAFO	–	10Co	T4–T12	$13.79 \pm 1.68$ (1.25%)	$67.39 \pm 65.98$ (2.14%)	$1.90 \pm 1.43$ (18.01%)	$10.44 \pm 1.47$ (2.04%)	$5.11 \pm 0.62$ (1.39%)
Kwon et al.^39^	KAFO	–	10Co	T4–T11	$11.80 \pm 1.80$ (− 13.36%)	–	–	–	4.37 * (− 13.29%)
Kawashima et al.^56^	ARGO	–	10Co	T5–T12	$18.20 \pm 3.80$ (33.63%)	–	–	–	
Winchester et al.^57^	RGO	–	2Co + 1In	T5–T10	$14.20 \pm 1.80$ (4.26%)	–	–	–	–
Bernardi et al.^58^	RGO	–	10	T4–T12	$13.30 \pm 3.70$ (− 2.35%)	–	–	–	–
Felici et al.^59^	RGO, ARGO	–	6Co	T5–L1	$14.30 \pm 4.70$ (4.99%)	–	–	–	–
Massucci et al.^60^	ARGO	–	6Co	T3–T12	$13.70 \pm 3.50$ (0.59%)	–	–	–	–
Ijzerman et al.^61^	ARGO	–	10Co	T4–T12	$17.60 \pm 2.00$ (29.22%)	–	–	–	–
Merati et al.^36^	HGO	–	4Co	C7–T8	$13.40 \pm 3.00$ (–1.62%)	–	–	–	–
	RGO	–	6Co	T3–T11	$13.80 \pm 3.50$ (1.32%)	–	–	–	–
Average of studies using mechanical orthoses	–	–	–	–	14.48 (6.31%)	–	–	–	4.37 (− 13.29%)

Values represent the mean and standard deviation. Values inside parentheses show the difference in percentage (%) with respect to the ABLE Exoskeleton

$^*$ Indicates that authors calculated the value.

$^1$ Change in $VO_{2}$ between resting and walking test.

$^2$ We set 1 MET as 2.7 ml/kg/min for participants with SCI^62^.

ARGO: Advanced reciprocating gait orthosis; C: Cervical; Co: motor-complete SCI; HGO: Hip guidance orthosis; In: motor-incomplete SCI; KAFO: Knee-ankle-foot orthosis; L: Lumbar; RGO: Reciprocating gait orthosis; T: Thoracic.

### Questionnaires

#### Psychoshocial impact

**Figure 6:**
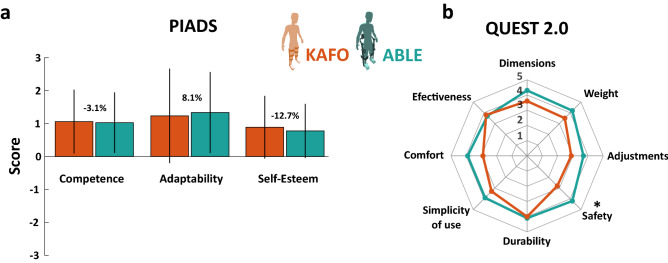
(**a**) Scores of the three subscales of the PIADS questionnaire for each device. Scores range from − 3 (maximum negative impact) to zero (no perceived impact) to +3 (maximum positive impact). (**b**) First 8 items of the QUEST 2.0 questionnaire for each device. These items assess characteristics of the corresponding device in terms of the shown dimensions. Answers are categorized on a five-point scale that ranges from 1 (not satisfied at all) to 5 (very satisfied). Stars (*) indicate statistically significant differences (*p* $< 0.05$).

Overall psychosocial impact measured by the PIADS was positive for both devices (KAFO: $27.20 \pm 26.98$; ABLE: $26.50 \pm 23.94$; *p* = 0.683; questionnaire score range: [− 78, 78]). Note that the standard deviation has the same size that the mean, which indicates a substantial variability across participant’s answers. The ABLE Exoskeleton showed overall positive scores for all the subscales (Fig. 6a): competence (KAFO $1.06 \pm 0.97$; ABLE: $1.03 \pm 0.92$; *p* = 0.837), adaptability (KAFO: 1.23 ± 1.43; ABLE: $1.33 \pm 1.23,$ *p* = 0.382), and self-esteem (KAFO: 0.89 ± 0.95; ABLE: $0.78 \pm 0.82,$ *p* = 0.573). However, no significant differences were found between devices.

#### User satisfaction

The results of the QUEST 2.0 showed that participants were, on average, satisfied with both devices for all the items (Fig. 6b). The ABLE Exoskeleton was considered to be significantly safer than the KAFOs and presented, in general, slightly better scores in the 8 items assessed: dimensions (KAFO: $3.60 \pm 0.70$; ABLE: $4.30 \pm 0.95$; *p* = 0.097), weight (KAFO: $3.50 \pm 1.08$; ABLE: $4.20 \pm 1.23$; *p* = 0.250), adjustments (KAFO: $2.90 \pm 1.10,$ ABLE: $3.70 \pm 1.25$; *p* = 0.066), safety (KAFO: $2.80 \pm 1.03,$ ABLE: $4.20 \pm 1.03$; *p* = 0.021), durability (KAFO: $4.00 \pm 0.82$; ABLE: $4.10 \pm 0.99$; *p* = 0.811), simplicity of use (KAFO: $3.30 \pm 1.25$; ABLE: 3.90 ± 1.37; *p* = 0.217), comfort (KAFO: $2.90 \pm 1.10$; ABLE: 3.90 1.20; *p* = 0.159), and effectiveness (KAFO: $3.80 \pm 1.03$; ABLE: 3.70 ± 1.42; *p* = 0.879).

## Discussion

This randomized, crossover clinical trial is, to the best of our knowledge, the first study that has been conducted to compare the use of a knee-powered lower limb exoskeleton against conventional KAFOs in people with motor-complete SCI, in terms of gait kinematics, spatiotemporal parameters, metabolic cost, and users’ satisfaction. The results support our first hypothesis that walking with the robotic device would improve gait kinematics and spatiotemporal parameters compared to walking with KAFOs. However, contrary to our second hypothesis, the improvement in gait kinematics using the exoskeleton did neither result in significant improvements in performance during the standardized gait tests, nor a significant reduction in energy consumption compared to walking with the KAFOs. Finally, in terms of users’ satisfaction, the ABLE Exoskeleton presented on average higher scores than the KAFOs.

This study used the three most common standardized clinical tests applied for evaluating gait in patients with neuromuscular impairments when walking with robotic devices (10 MWT, TUG, and 6 MWT)^41^. Although, none of the clinical test metrics (distance for the 6 MWT, time for the TUG, and gait speed for the 10 MWT) showed significant differences between devices, we found differences in terms of the correlation between LOI and the three clinical test metrics (see Fig. 2). While significant, strong correlations were found between LOI and clinical test metrics when walking with the KAFOs, these correlations faded when walking with the ABLE Exoskeleton. This result suggests that walking with the ABLE Exoskeleton is less conditioned by the LOI of the user compared to walking with KAFOs. The higher uniformity of the clinical outcomes obtained among different LOI when using the robotic device may be attributed to the stability offered by the lumbar module, which reduces the limitations that the SCI causes on trunk and hip control, especially in higher LOI.

When comparing the results of the clinical test metrics obtained in the present study with the ABLE Exoskeleton (which only has knee actuation) with commercially available exoskeletons intended for overground walking in people with SCI (which have both knee and hip actuation), we found that the results with the ABLE Exoskeleton were within the range of other exoskeletons for the three clinical test metrics that were used: distance covered during the 6 MWT (ABLE: $47.13 \pm 29.94$ m; range of means from other exoskeletons: [39.1, 99.1] m)^32,39,63–65^, gait speed during the 10 MWT (ABLE: $0.17 \pm 0.10$ m/s; range of means from other exoskeletons: [0.16, 0.31] m/s)^63,64,66^, and time of the TUG test (ABLE: $102.70 \pm 52.68$ s; other exoskeletons: 100.5 ± 29.90 s)^65^. These comparisons need to be interpreted with caution because, in contrast to the ABLE Exoskeleton, the other exoskeletons have an active hip joint, which can assist in providing trunk stability and forward propulsion during gait. Also note that for this comparison, we selected studies with participants that had similar characteristics to the ones of the present study (i.e., chronic participants with motor-complete paraplegia due to SCI).

Regarding the analysis of the spatiotemporal variables, we found that step length was significantly increased when using the ABLE Exoskeleton. This increase in step length could have been promoted by the significantly higher range of motion of the thigh angle in the sagittal plane (see Fig. 3c). In this regard, it is important to point out that this angular difference is not related to the use of the Walkabout joint when walking with KAFOs. While it is true that this joint may constrain the thigh ROM due to a mechanical limit, no participants reached that limit during the tests. Additionally, cadence and duration of the gait phases did not present significant differences between devices. The values of the spatiotemporal variables found in this study with both devices are still far from the averages of unimpaired adults^67^ (cadence: 109.80 steps/min, step length: 25.60% BH, swing time: 37.36%).

Regarding the analysis of the gait kinematics, we found significant improvements in terms of compensatory movements and weight shifting when walking with the ABLE Exoskeleton compared with the KAFOs. Firstly, in regards to the compensatory movements, we found that participants had significantly less circumduction motion when walking with the ABLE Exoskeleton compared to the KAFOs (see Fig. 4a,b), thus promoting a more physiological gait pattern. This result is consistent with what was initially expected, since the passive hip joint of the ABLE Exoskeleton restricts rotations out of the sagittal plane, and the knee actuator provides flexion-extension assistance during the swing phase, which promotes foot clearance. In contrast, the lack of knee flexion in the KAFOs forces the user to perform circumduction movements (and probably other compensatory movements) to achieve enough foot clearance and flex the hip when performing a step. Performing these circumduction movements has been shown to result in excessive loads onto the upper limb joints^68,69^ and increased joint pain risk^25^.

Secondly, in regards to the weight shifting, the relative position of the PCOM with respect to the leading foot at toe-off was significantly closer when using the robotic device, suggesting a better weight distribution at toe-off (see Fig. 4c,d). This improvement is most probably related to the reduction of the aforementioned compensatory movements, together with the restriction of out of sagittal plane hip rotations and the knee assistance provided by the ABLE Exoskeleton. Proper weight distribution is particularly important in gait rehabilitation, since the ability to control weight shifting is a key component of a natural gait pattern and a prerequisite for independent walking^70,71^. In fact, inadequate weight shifting may impede an efficient leg swing, inducing a shorter step length and slower gait speed. Interestingly, the difference between devices in terms of position of the PCOM relative to the leading foot was significant for the mediolateral axis, but not for the anteroposterior axis. A reason for that may be the high variability of the anteroposterior position of the PCOM at toe-off when walking with KAFOs, which presented undesirable positive position values with respect to the leading foot (i.e., the PCOM overpassed the leading foot and thus was placed outside of the legs base of support). This position of the PCOM indicates excessive body tilting in the anterior direction, which requires effort from the upper body to keep in balance. Consequently, although the average relative position of the PCOM with respect to the leading foot is small when using the KAFOs, 25% of the participants shown positive position values, which implies that the weight distribution is in general not ideal for the above-mentioned reasons.

Regarding the analysis of the metabolic cost of walking, we found that, contrary to our hypothesis, the aforementioned improvements in gait kinematics when walking with the ABLE Exoskeleton did not result in a reduction in the metabolic cost compared to walking with KAFOs (see Fig. 5). There are four reasons that can explain why no differences were identified in terms of energy expenditure between both devices.

Firstly, it is well known that adding mass to the legs increases the metabolic cost of walking^72,73^. Therefore, we think that despite the fact that participants had a more natural walking pattern when walking with the ABLE Exoskeleton, the considerably higher mass of the robotic device compared to the KAFOs (KAFO: 3.16 kg on average vs. ABLE Exoskeleton: 9.80 kg) caused a higher metabolic penalty. In fact, the estimated metabolic penalty, based on coefficients from the literature^73^, is 1.65 ml/kg/min higher with the robotic device (KAFO: 0.82 ml/kg/min; ABLE: 2.47 ml/kg/min), which represents an $8.12 \pm 1.45$ $\%VO_{2peak}$ (KAFO: $4.06 \pm 0.72$ $\%VO_{2peak}$; ABLE: 12.18 ± 2.18 $\%VO_{2peak}$).

Secondly, it is noteworthy that all previous studies that compared KAFOs and robotic exoskeletons in people with SCI reported significant improvements in energy efficiency when walking with the exoskeletons^26,37–39^, yet all of them used robotic devices that provided powered assistance to both hip and knee joints. In contrast, the ABLE Exoskeleton device used in the present study only provided powered knee assistance. According to the study by Farris and Sawicki^42^, the positive mechanical power during normal walking is largely delivered by the ankle and hip joints, while the knee joint has a considerably lower contribution. In addition, it has been shown that assisting only the knee joint with an exoskeleton during walking in able-bodied subjects produced the smallest reduction in metabolic cost compared to providing hip-only and ankle-only assistance, and this reduction was not statistically significant when compared to the unpowered condition^74^. Therefore, we believe that this low positive-power contribution of the knee joint may also partly explain why no significant reductions in metabolic cost were found when walking with the ABLE Exoskeleton compared to walking with KAFOs. Nevertheless, it is worth mentioning that training with the ABLE Exoskeleton met the recommendations of exercise intensity for people with SCI (i.e, intensity of 50–80 $\%VO_{2peak}$)^75^.

Thirdly, the lack of trunk stability (i.e., the ability to keep the trunk stable and to return to a stable state after some perturbation^76,77^) in the sagittal plane is another reason that may explain the similarities in metabolic cost between devices. Even though the lumbar module of the ABLE Exoskeleton passively restricted hip joint motions in the frontal and transverse planes, participants still had free hip flexion-extension in the sagittal plane in a similar way as with the KAFOs, thus leading to the mentioned difficulties for keeping body stability in dynamic conditions while walking. This same reason could also explain the differences in metabolic cost when comparing the ABLE Exoskeleton with other exoskeletons in the literature (see Table 2), since trunk stability assistance is provided with hip-powered actuators.

Finally, the unbalanced experience level of the study participants using both devices could have biased the results on the metabolic cost. All participants in this study were chronic patients with years of experience using KAFOs. Yet, only three participants had previously walked with other powered wearable exoskeletons. Therefore, in general, participants had less time to learn to use the ABLE Exoskeleton than the KAFOs. The study by Chantraine et al.^78^ investigated the relation between expertise and energy expenditure in paraplegic patients with complete SCI when walking with long leg braces. The metabolic cost was measured during walking and compared the energy consumption between unaccustomed and accustomed patients using braces. They found that in the unaccustomed group, the speed of walking was slower and with a significantly higher energy consumption than the accustomed group. Moreover, the energy consumption was inversely related to the regular use of long leg braces. Therefore, we believe that another of the main causes for the higher metabolic cost observed in the participants of our study when walking with the exoskeleton was due to their low experience level.

Regarding the comparison of our results on metabolic cost with other studies that also measured metabolic cost during exoskeleton-assisted walking (Table 2), we found that all previous studies reported a higher energy efficiency. However, all these works used wearable exoskeletons with, at least, knee and hip powered actuation. This result suggests that knee-only powered assistance is not sufficient to reduce the metabolic cost of walking compared to KAFOs, and active assistance of the hip or ankle joints seems necessary. Interestingly, we also found that the $VO_{2avg}$ when walking with passive orthoses from previous studies was on average 5% higher than the $VO_{2avg}$ measured when walking with KAFOs in the present study. This contrasts with the fact that in almost all the previous studies (7 out of 8) passive orthoses with a lumbar module (e.g., RGO: reciprocating gait orthoses) were used, even though they are considered to reduce overall metabolic cost compared to KAFOs^79,80^. However, the comparison among studies must be taken with caution, as the primary metric used by the majority of them was the $VO_{2avg}$. The $VO_{2}$ of an individual is dependent on several factors, such as genetics^81,82^, gender^83^, age^84^, body composition^85,86^, and/or fitness^87^. Thus, the comparison of these data may be biased and further studies should provide normalized metrics (e.g., $\%VO_{2peak}$) for a proper comparison of the results.

Lastly, regarding the evaluation of users’ satisfaction with the assistive devices, the ABLE Exoskeleton was considered significantly safer than the KAFOs, and was evaluated as more comfortable and easier to use and adjust than the KAFOs (see Fig. 6b). These results contrast with the study by Kwon et al.^39^ where, despite the higher energy efficiency achieved with the ReWalk Exoskeleton, the robotic device was not superior to KAFOs in terms of safety, effects, efficiency, usability, and satisfaction. Although very few studies have focused on evaluating users’ satisfaction with gait assistive devices, we consider that these types of studies are crucial to better understand how users perceive technology and provide guidance for future developments. With regards to the impact that the devices had on the functional independence, well-being, and quality of life, using the ABLE Exoskeleton was considered as good as a conventional KAFOs (see Fig. 6a).

The present study had some limitations. The inability to collect pelvis, trunk, and upper limb kinematics, due to the space constraints in the hospital and the set up of the optical cameras, was a limitation for a complete biomechanical analysis of compensatory movements. In line with this, the location of the markers on top of the assistive devices (KAFOs and ABLE Exoskeleton) may also have added slight errors due to the relative movement between the participants’ lower limbs and the devices. Also, for a more comprehensive biomechanical analysis, in addition to kinematic outcomes future studies should consider the use of instrumented insoles and walker to accurately analyze the weight distribution between the lower limbs and the walker. Moreover, the length of the pathway (10 m) for the 6 MWT was short and forced participants to spend time turning around. Furthermore, while it is interesting to have a wide range of injury levels among the study participants to evaluate the assistive devices, this diversity might have added a larger variability to the data that could have limited the statistical significance of the results. In like manner, the differences in the LoA between devices, despite not being statistically significant, might have confounded the comparison. Finally, learning to use an exoskeleton is time-consuming and varies among users^88^, making it difficult to define the number of training sessions needed to use it independently and efficiently. We recommend that future studies consider a larger amount of training sessions to allow participants to manage the robotic device more comfortably. Future work will focus on understanding the user’s learning process of operating the ABLE Exoskeleton.

In conclusion, the present study was the first to compare the use of KAFOs (standard of care) against a knee-powered lower limb exoskeleton (i.e., the ABLE Exoskeleton) for assisting gait in people with motor complete SCI. Our findings suggest that using a knee-powered exoskeleton improves gait kinematics in people with SCI. The knee flexion assistance, together with having a lumbar module that constrains hip movements out of the sagittal plane, enabled the participants to walk with a more physiological gait pattern (less compensatory movements, better weight shifting, and longer step length) using the ABLE Exoskeleton compared to using the KAFOs. However, the improvements in gait kinematics did not extend to significant improvements in energy efficiency. Probably the low contribution of the knee joint on the metabolic cost of walking, together with the lack of trunk stability in the sagittal plane, were not enough to reduce the effort that ambulation entails in people with complete SCI. Thus, the results of this study suggest that to significantly reduce the energy consumption required by people with SCI to walk with KAFOs, providing only active knee assistance is insufficient, and active assistance of the hip or ankle joints is necessary. In terms of user satisfaction, the ABLE Exoskeleton was considered significantly safer and presented on average higher scores than the KAFOs. Finally, the insights provided by this clinical trial have been key for the engineers at ABLE Human Motion to develop the next version of the ABLE Exoskeleton. Specifically, improvements will be made through the addition of hip actuation to reduce the metabolic cost of the users, and the increase of the support provided by the lumbar module to promote better trunk stability. Clinical studies will be performed in the future with this new design.

## Supplementary Information

Supplementary Information 1.

Supplementary Information 2.

## Data Availability

The datasets used and/or analysed during the current study are available from the corresponding author on reasonable request.
