# Supplementary material for: Comparing walking with knee-ankle-foot orthoses and a knee-powered exoskeleton after spinal cord injury: a randomized, crossover clinical trial
Source: Sci Rep. 2022 Nov 9;12:19150. doi: 10.1038/s41598-022-23556-4 (PMC9646697; doi:10.1038/s41598-022-23556-4)
Supplement: Supplementary file 1 — Supplementary Information 1. [file 41598_2022_23556_MOESM1_ESM.pdf]

Supplementary Material to:

Comparing Walking with Knee-Ankle-Foot Orthoses and a Knee-Powered Exoskeleton After Spinal Cord Injury: A Randomized, Crossover Clinical Trial

A. Rodríguez-Fernández, J. Lobo-Prat, R. Tarragó, D. Chaverri, X. Iglesias, L. Guirao-Cano, J.M. Font-Llagunes

Supplementary Figure 1: CONSORT flow diagram

Supplementary Table 1: Inclusion/Exclusion criteria

Supplementary Table 2: ABLE Exoskeleton step parameters.

Supplementary Table 3: Results.

Supplementary Figure 2: Level of assistance (LOA).

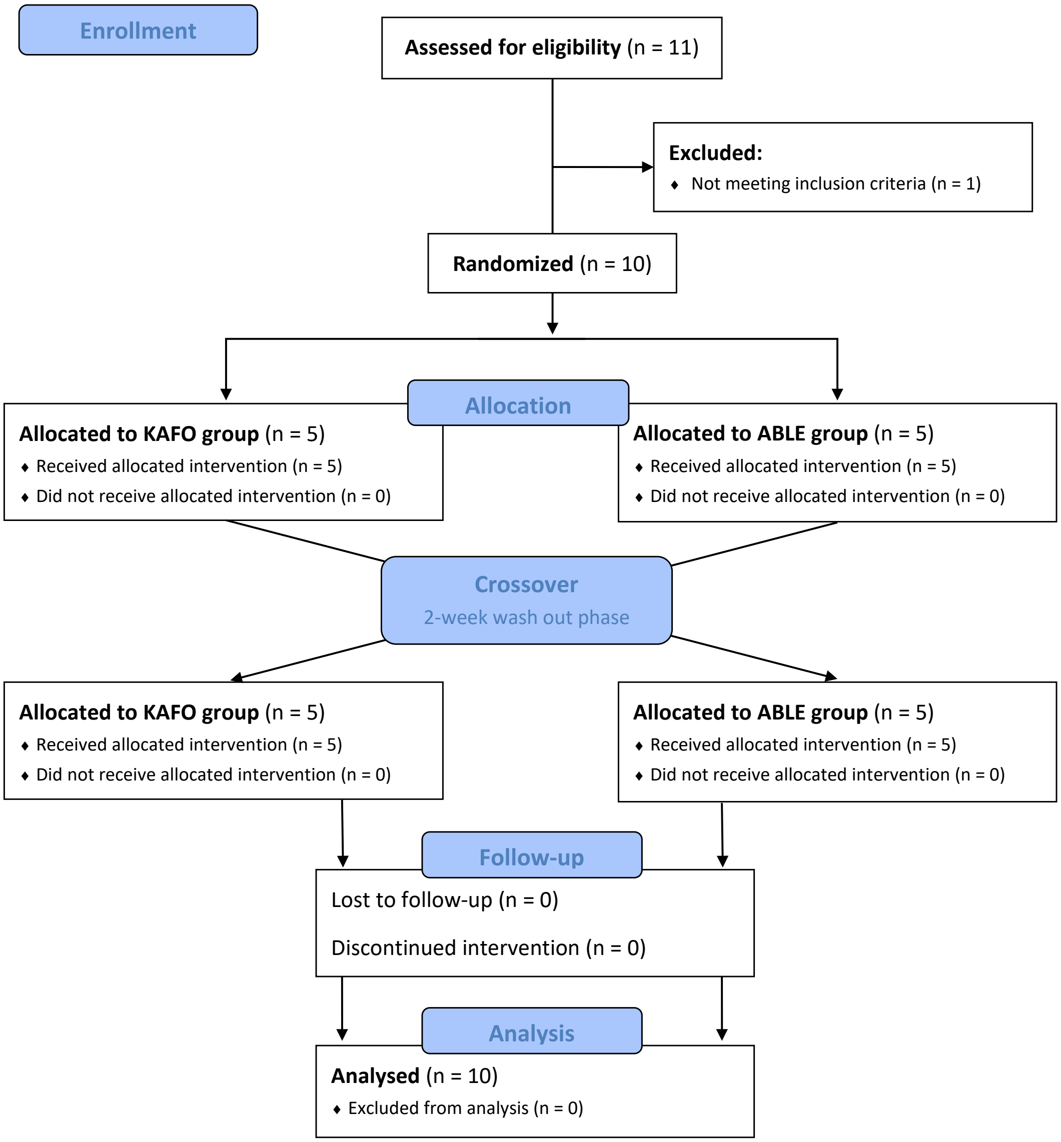

Supplementary Figure 1. CONSORT flow diagram

Inclusion Criteria

- Between 18 and 70 years old
- Chronic or subacute SCI
- Currently as an inpatient or outpatient at the participating hospital
- AIS A to C
- Previous experience walking with KAFO
- Capable to give personal consent

Exclusion Criteria

- WISCII >16 without exoskeleton
- 5 or more risk factors of bone fragility*
- Fragility fractures of the lower limbs in the last 2 years
- Deterioration >3 in the International Standards for Neurological Classification of SCI (ISNCSCI) score in the last 4 weeks
- Spinal instability
- Modified Ashworth Scale (MAS) >3 in lower limbs
- Inability to tolerate 30 minutes of standing without clinical symptoms of orthostatic hypotension
- Inability to walk 5 meters with KAFO and the help of a walker
- Psychological or cognitive issues that do not allow a participant to follow study procedures
- Any neurological condition other than SCI
- Medically unstable
- Severe comorbidities, including any condition that a physician deems inappropriate for completing study participation
- Skin problems
- Height, width, weight, or other anatomical limitations (such as differences in leg length) incompatible with the device
- Insufficient joint range of motion (ROM) for the device
- Known pregnancy or lactation

Supplementary Table 1. Inclusion/Exclusion criteria

** Craven, B., Robertson, L., McGillivary, C. & Adachi, J. Detection and Treatment of Sublesional Osteoporosis Among Patients with Chronic Spinal Cord Injury: Proposed Paradigms. Top Spinal Cord Inj Rehabil 4, 1–22 (2009).*

Patient	Peak knee flexion (º)	Swing time (s)	Flexion-extension ratio
**P1**	63	1.1	-8
**P2**	58	1.1	-9
**P3**	60	1.0	6
**P4**	60	0.9	-3
**P5**	64	1.0	-4
**P6**	64	1.1	-7
**P7**	53	1.0	-10
**P8**	60	1.2	-9
**P9**	53	0.8	-4
**P10**	53	1.0	-8
**Mean ± SD**	58.80 ± 4.44	1.02 ± 0.11	-5.6 ± 4.74

Supplementary Table 2. ABLE Exoskeleton step parameters. The shape of the ABLE Exoskeleton knee angular trajectory during the swing phase can be adapted to the participant's requirements through the following 3 parameters: (1) The peak knee flexion (allowed range: from 40 to 80º) establishes the maximum knee flexion angle that the actuator reaches during the swing phase (0º means the knee is completely straight), (2) the swing time (allowed range: from 0.5 to 2.5 s) defines the duration of the swing phase, and (3) the flexion-extension time ratio (allowed range: from -2 to 2) determines the time displacement of the peak knee flexion (i.e., defines the time from the starting point of the swing phase to the point where the peak knee flexion is reached). A negative value in the flexion-extension time ratio means that the flexion time is shorter than the extension time, and vice versa. Note that a minimum time between the starting point, or final point, of the swing phase and the position of the peak knee flexion is required for the actuator to get the desired position.

Supplementary Table 3. Results from all the metrics shown in the study.

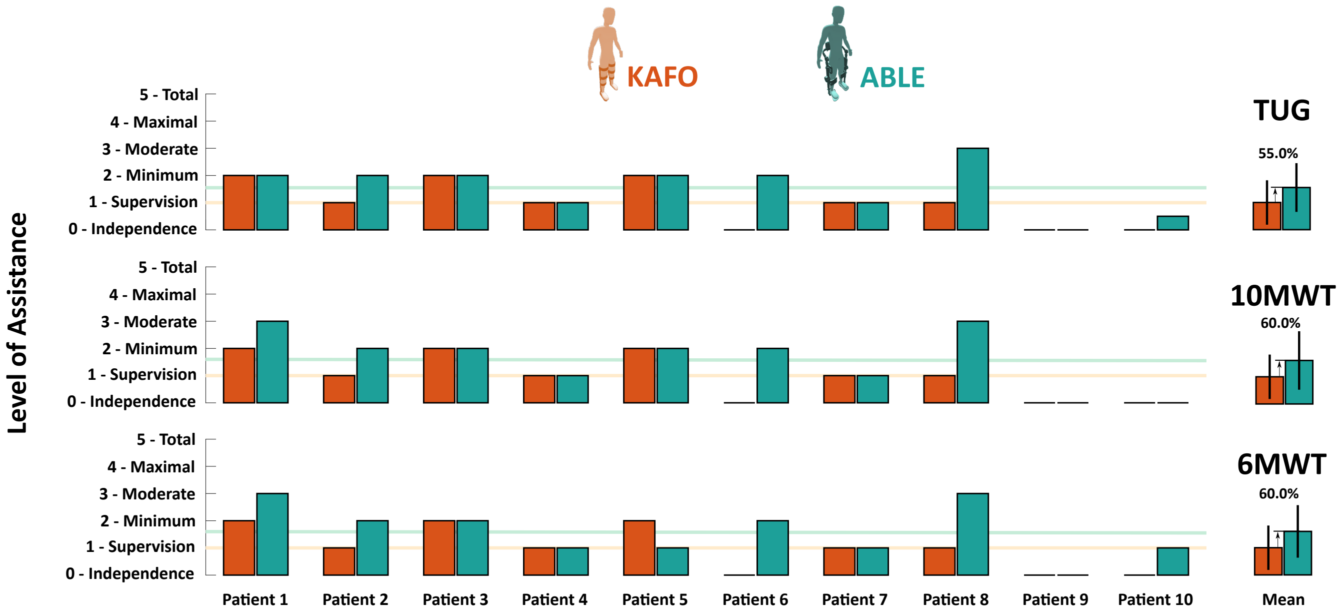

Supplementary Figure 2. Level of assistance for each participant in the TUG, 10MWT, and the 6MWT (from top to bottom). The level of assistance was modified from the FIM as follows: *Total* assistance (participant provides 0%-24% of effort required to walk with the device), *Maximal* assistance (participant provides 25%-49% of effort required to walk with the device), *Moderate* assistance (participant provides 50%-74% of effort required to walk with the device), *Mininimal* assistance (participant provides 75% or more of effort required to walk with the device, but less than 100%), *Supervision* (participant provides 100% of effort; helper maintains touch or near-touch contact, but provides no assistance), *Independence* (no supervision needed). Modified FIM values where scaled from 0 to 5, where 0 means *Independence* and 5 *Total Assistance*. Horizontal lines indicate the mean of all the participants (TUG, KAFO: 1 ± 0.82; ABLE: 1.55 ± 0.97; $p = 0.098$; 10MWT, KAFO: 1 ± 0.82; ABLE: 1.60 ± 1.07; $p = 0.095$; 6MWT, KAFO: 1 ± 0.82; ABLE: 1.60 ± 0.97; $p = 0.081$).
